# Psychometric properties of the oral feeding assessment in premature infants scale

**DOI:** 10.1038/s41598-022-11521-0

**Published:** 2022-05-12

**Authors:** Sergio Alonso-Fernández, Carlos Rodrigo Gonzalo de Liria, Teresa Lluch-Canut, Laura Poch-Pla, Josep Perapoch-López, Maria-Eulàlia Juvé-Udina, Maria-Antonia Martínez-Momblan, Bárbara Hurtado-Pardos, Juan-Francisco Roldán-Merino

**Affiliations:** 1 Faculty of Nursing, Rovira i Virgili University, Avinguda Catalunya, 35, 43002 Tarragona, Spain; 2 Health Sciences Research Institute and University Hospital Germans Trias i Pujol, Ctra de Canyet s/n, 08916 Badalona, Spain; 3 IDIBELL, Bellvitge Biomedical Research Institute, Avinguda de la Granvia, 199, L’Hospitalet de Llobregat, 08908 Barcelona, Spain; 4 Department of Pediatrics, Germans Trias i Pujol University Hospital, Ctra de Canyet s/n, 08916 Badalona, Spain; 5 Faculty of Medicine, Universitat Autònoma de Barcelona, Ctra Can Ruti-Camí Escoles, s/n, 08916 Badalona, Spain; 6 Department of Psychosocial and Mental Health Nursing, School of Nursing, Faculty of Medicine and Health Sciences, University of Barcelona, Carrer de la Feixa Llarga, s/n, L’Hospitalet de Llobregat, 08907 Barcelona, Spain; 7 Neonatal and Paediatric Intensive Care Unit, Doctor Josep Trueta University Hospital, 17007 Girona, Spain; 8 Nursing Research Group (GRIN), IDIBELL Bellvitge Biomedical Research Institute, Avinguda de La Granvia, 199, L’Hospitalet de Llobregat, 08908 Barcelona, Spain; 9 Fundamental Care and Medical-Surgical Nursing Department, School of Nursing, University of Barcelona, Pavelló de Govern, 3° pl, L’Hospitalet de Llobregat, 08907 Barcelona, Spain; 10 Biomedical Research Networking Centre of Rare Diseases (CIBER-ER), Unit 747 ISCIII, Madrid, Spain; 11 Campus Docent Sant Joan de Déu, School of Nursing, University of Barcelona, Carrer Miret i Sans 10-16, 08034 Barcelona, Spain

**Keywords:** Neonatology, Preterm birth

## Abstract

Professionals that work in neonatal units need to identify the strengths and weaknesses of the premature infant who is in the transition process from feeding through a gastric tube to oral feeding. The main aim of this study was to validate the Oral FEeding Assessment in premaTure INfants (OFEATINg) instrument. A psychometric validity and reliability study was conducted in Neonatal Intensive Care Units of two public, metropolitan, university hospitals. The study population were premature infants at a postconceptional age of 31–35 weeks. The study included evaluation of the reliability, convergent, discriminant and construct validity, sensitivity and specificity of the OFEATINg instrument. A total of 621 feedings of 56 preterm infants were evaluated. Confirmatory factor analysis identified 3 factors and 13 indicators with a good fit to the model. Cronbach's alpha coefficient was 0.78. The instrument showed high indices of inter-rater reliability (Pearson 0.9 and intraclass correlation coefficient 0.95). The OFEATINg scale is a valid and reliable instrument for evaluating the readiness for oral feeding of preterm infants. It may enable clinicians to evaluate the physiological and behavioral abilities involved in the oral feeding process and help them make decisions related to the transition to full oral feeding.

**Clinical trial registration:** This study was prospectively registered at the two Institutional review boards.

## Introduction

The Oral FEeding Assessment in premaTure Infants (OFEATINg) was recently designed and may help clinicians to evaluate oral feeding readiness and oral feeding success, defined as the infant’s ability to maintain physiologic stability and meet the combined criteria of feeding proficiency (≥ 30% of the prescribed volume during the first 5 min), feeding efficiency (≥ 1.5 mL/min over the entire feeding), and intake quantity (≥ 80% of the prescribed volume)^1^. Despite this, its psychometric properties have not yet been studied. The purpose of this study was to validate an instrument to identify oral feeding skills in premature infants admitted in a Neonatal Intensive Care Unit (NICU).

## Literature review

Historically, premature infants (PI) were discharged only when they achieved a certain weight. However, randomized clinical trials have shown that an earlier discharge is possible without adverse health effects when discharge decision is based on physiologic criteria rather than body weight. These criteria are usually based on parental care skills and aspects of development^2^. When evaluating an infant’s readiness for oral feeding, physiological and behavioral parameters are more important than maturity^3^. The interaction between multiple physiological systems, behavioral dynamics, and social interactions make infant feeding a complex, dynamic system^4,5^.

Several instruments have been designed to describe and measure infant oral feeding skills. While some instruments were designed to evaluate oral breastfeeding (Infant Breastfeeding Assessment Tool (IBFAT)^6^, Systematic Assessment of the Infant at Breast (SAIB)^7^, Mother-Baby Assessment (MBA)^8^, LATCH^9^, Preterm Infant Breastfeeding Behaviour Scale (PIBBS)^10^, Preterm Oral Feeding Readiness Assessment Scale (POFRAS^11^), others were designed to evaluate only oral bottle feeding (Preterm Infant Nipple Feeding Readiness Scale (PINFRS)^12^, Early Feeding Skills Assessment (EFS)^13^. The Neonatal Oral-Motor Assessment Scale (NOMAS) is the only one who evaluates both, but was only designed to assess biomechanical components for successful feeding, without including behavioural aspects^14^. When clinicians are evaluating human responses using scales or questionnaires, the measurements of these variables (for example, oral feeding readiness) are dependent upon their definitions. Moreover, they may vary from one person to another and the way they are measured. Thus, as the determinants of human behavior are far from perfect, the measurements of the instruments designed will have to be validated (tested) against actual performance^15^.

A review of the feeding assessment tools showed that the NOMAS was the instrument that has been examined more thoroughly and showed more consistent results in psychometric properties than the others. Also, they found that there was limited evidence of psychometric properties for the EFS, MBA and SAIB. They concluded that some of the instruments have not been subjected to reliability and validity testing, or the validity studies published were performed with small sample sizes and limited sample representations^16^. In 2018, the EFS scale psychometric properties were evaluated and show good reliability (cronbach’s alpha: 0.81) and good contruct validity. The latest version consists of 19 items^17^.

Finally, many of these instruments have not been validated in Spanish. At present, the LATCH is the only instrument that has been adapted and validated in Spanish, although it only evaluates feeding during breastfeeding.

Feeding methods have also been developed in which the caregiver is guided by the responses of the preterm infant. Methods such as the Supporting Oral Feeding in Fragile Infants (SOFFI)^18–20^ or the Infant-Driven Feeding Scales IDFS^21^ are applied as decision-making algorithms and include both behavioral responses and caregiver interventions in their assessments. The impact of implementing these methods has also recently been evaluated^22,23^. In our context, many NICUs are implementing these methods. But the problem to find a valid and reliable instrument to evaluate oral feeding remains. Healthcare professionals need validated instruments in Spanish that allow them to identify the strengths and weaknesses of the PI who is in the process of transition from gastric tube feeding to oral feeding. It is necessary to design a new scale with fewer items, based on physiological and behavioral aspects, that evaluates breastfeeding and bottle feeding, for premature infants, and adapted to our healthcare reality and our language.

## Methods

In Spanish NICUs, the use of valid and reliable tools to assess oral feeding in premature infants is a problem. Professionals need tools validated in Spanish that enable them to identify the strengths and weaknesses of the PTNB who is in the transition process from feeding through a gastric tube to oral feeding. The design of a new scale is needed with fewer items, based on physiological and behavioural, which evaluates breastfeeding and bottle feeding, for newborns of any gestational age, and adapted to our healthcare reality and language.

### Design

Psychometric validity and reliability study.

### Study setting

The setting for data collection were two level III NICUs of two university hospitals. They have an intensive care area for the care of 5 and 8 patients, and an intermediate care area 10 and 18 patients. During 2016 a total of 254 and 315 premature infants were admitted.

### Participants and sample size calculation

The participants were PIs in the NICUs of the two hospitals. Infants were included in the study if they had a postconceptional age of 31–35 weeks. Infants were excluded if they had an abdominal pathology that had been surgically corrected, had undergone major surgery or had a severe neurological disorder. The participants were recruited consecutively between May 2016 and March 2017.

### Instruments

The Oral FEeding Assessment in premaTure INfants (OFEATINg), known in Spanish as Valoración de la ALimentación Oral en PREMaturos (VALOPREM) was designed to evaluate readiness to begin a feed and evaluation of a feeding event. It consists of 13 items grouped into 3 factors that evaluate different aspects related to feeding: “Capacity to coordinate sucking-swallowing-breathing” (six items: 3, 4, 5, 6, 7 and 11), “Capacity to administer oxygen reserves” (four items: 8, 9, 12 and 13) and “Capacity to take the teat or nipple” (three items: 1, 2 and 10). First ten items are evaluated during oral feeding, with the remaining three items being assessed thirty minutes after feeding completion. There are four Likert-style responses for each item, with values ranging from 1 to 4, with a higher score reflecting greater readiness for oral feeding. The scores for items 4, 8, 9 and 12 must be inverted (1 = 4, 2 = 3, 3 = 2, 4 = 1) before calculating the total score. The total score of the OFEATINg scale is the sum of the scores of all the items, with a minimum score of 13 and a maximum of 52.

### Ethical considerations

The study was conducted in accordance with the principles of the Declaration of Helsinki. The survey ensured the anonymity and confidentiality of the subjects and the data gathered. Informed consent of the parents or legal guardians of the participants was obtained. The study was approved by the Clinical Research Ethics Committee of the participating hospitals (PI-15-014 and 2016.118).

### Data collection

All nurses were taught how to administer the scale through in-service seminars. The seminars were in the format of 40 min of oral presentation and 20 additional minutes to present the study and the scale and to raise questions or doubts. The contents of the session dealt with the protocol for transition to oral feeding, how to recognize clinical signs of readiness or no tolerance of bottle or breastfeeding, and difficulties or reasons to decide to suspend the feeding. The purpose of the study and how to administer the scale was also presented. Two training sessions per shift were given to all the nurses and staff, as well as two additional sessions for all the pediatricians and neonatologists. All the staff assisted to any of the 10 sessions planned.

Clinical staff offered all oral feedings and decided to maintain, interrupt, or stop oral feeding according to the clinical signs identified in the previous training sessions (nipple refusal, coughing, choking, vomiting, signs of respiratory effort (intercostal retractions, nasal flaring, retractions, etc.). The reasons to stop or interrupt the feeding were also reported on the data collection form. The OFEATINg scale was administered to each infant once a day.

The feeding evaluations for inter-rater reliability were evaluated independently by two nurses with more than five years of professional experience in NICUs. During twice evaluations, both nurses observed the same feed intake, but the recording in the document was done in different boxes and in a confidential manner.

### Data analysis

The provision for oral feeding in a preterm infant may vary from day to day, so each assessment of oral feeding has been analyzed independently. A required sample size of at least 325 evaluations was calculated, with an alpha of 0.05, to detect a minimum Cronbach’s alpha coefficient of 0.7 at a confidence level of 95%^24^. It was decided to include double the estimated sample size (n = 650) to conduct exploratory factor analysis and confirmatory.

The instrument was analyzed with the following tests: convergent validity (Pearson correlation coefficient), discriminant validity (Student T-test) and construct validity, through exploratory and confirmatory factor analysis. Reliability was assessed by analyzing the internal consistency and inter-rater agreement. The instrument's sensitivity and specificity were also analyzed.

**Figure 1:**
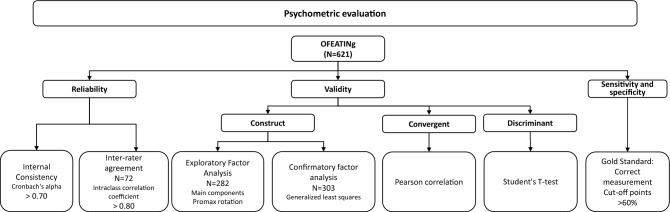
Psychometric testing process.

A descriptive analysis of all the variables included in the study was conducted. To analyze the reliability of the OFEATINg scale, the internal consistency was calculated using the Cronbach's alpha^25^. The inter-rater agreement was analyzed using the Intra-class Correlation Coefficient (ICC)^26^. To assess the construct validity, the sample was randomly divided into two subsamples^27^. An exploratory factor analysis (EFA) was conducted with the first subsample while a confirmatory factor analysis (CFA) was carried out on the second subsample. The EFA was conducted using the unweighted least squares method with oblique promax rotation on the first subsample (282 feeds). Beforehand, the significance of the EFA was verified using the Kaiser–Meyer–Olkin test (KMO) and the Bartlett sphericity test^28^. To select the number of factors, the recommendations of Kaiser–Guttman^29^ were followed, extracting the factors with eigenvalues greater than 1. The second subsample of 303 feeds was used for the CFA. To estimate the model parameters, the Generalized Least Squares (GLS) method was used. The GFI (Goodness of Fit Index) and RMSE (Root Mean Standard Error) absolute fit indices were calculated^30^. With respect to incremental fit indices, the AGFI (Adjusted Goodness of Fit Index), BBNFI (Bentler Bonnet Normed Fit Index) and BBNNFI (Bentler Bonnet Non-Normed Fit Index) indices were used. The parsimony indices related the fit achieved to the number of free parameters in the model. The normalized Chi-squared test was used, defined as the ratio between the Chi-squared value and the number of degrees of freedom^31^. The convergent validity was determined by analyzing the Pearson correlation coefficient between the total scale score and the scores for each of the subscales^32^. The discriminant validity was analyzed using Fisher’s exact test depending on whether or not the infant had fed correctly (feeding tolerance). We defined correct feeding as a feeding in which there were no signs that would have led to the suspension or interruption of oral feeding. In addition, the sensitivity and specificity were evaluated. The gold standard was considered to be when the infant completed the feed correctly. To set a good borderline point, a ROC (Receiver Operating Characteristic) curve was built. The cut-off points were also distributed to select the ones with sensitivity and specificity higher than 60%^33^. IBM SPSS v.22 and EQS v.6.1. software was used for the data analysis. A significance level of 5% was set. The entire psychometric testing process is summarized in Fig. 1.

### Ethical approval

Ethical approval was provided by the Institutional Review Board of [Germans Trias I Pujol University Hospital (Registry number PI-15-014) and Dr Josep Trueta University Hospital (registry number 2016.118) in advance of implementation. Written informed consent was obtained from the patients/guardians.

## Results

### Participants

The feedings of 56 preterm infants were evaluated. Nine participants were not included in the final analysis due to incomplete records. The average gestational age at birth was 31.30 ± 2.20 (25–34) weeks of gestation. The mean chronologic age at the first oral feed was 23.79 ± 17.26 (0–82) days. 51.9% of the infants were male. The birth weight was 1615 ± 499 (790–3180) grams. 49.1% were fed parenterally during the first days of life, which, by protocol, implied an absolute diet with a minimum amount of milk (0.5–2 ml) supplied through a gastric tube every three hours. In this group, the mean chronologic age at the first oral feed was 27 ± 16.04 (13–74). The total number of feeds evaluated was 585 (27.2 % breastfeeding). Each infant received 10.44 evaluations during NICU stay.

### Item analysis

Table 1 shows the values for the central tendency and variability measurements for each of the 13 items on the scale. The average total score of the survey was 44.40 ± 4.90 (29–52), with a median of 45.

**Table 1:** Descriptive statistics of items and internal consistency coefficient (Cronbach’s alpha) for the OFEATINg scale.

Content of the summarized items	Measurements of central tendency and variability					Cronbach’s alpha		
	Mean	SD	Median	Kurtosis	Asymmetry	Sub-scale total	Sub-scale total without item	Scale total without item
**Capacity to take the teat or nipple**						0.81		
1. At the start of feeding, when the teat or nipple rubs the baby's lips, they open their mouth	3.38	0.70	3	0.53	− 0.95		0.66	0.76
2. When approaching the teat/nipple, the tongue lowers to take it in	3.37	0.70	3	0.45	− 0.90		0.65	0.76
10. Is the newborn able to latch on to the teat/nipple?	3.05	0.64	3	0.35	− 0.33		0.88	0.77
**Capacity to coordinate sucking–swallowing–breathing**						0.77		
3. Calm swallowing	3.16	0.75	3	0.08	− 0.64		0.71	0.75
4. The newborn loses milk while feeding	3.37	0.72	3	1.01	− 1.06		0.77	0.77
5. The newborn stops sucking by themselves to breathe. It is not necessary for the person feeding them to do so	3.28	0.82	3	− 0.19	− 0.85		0.71	0.75
6. Rhythmic sequence and suction fluid	3.10	0.78	3	− 0.21	− 0.54		0.71	0.74
7. Easy breathing, respiratory work is not increased	3.11	0.88	3	− 0.32	− 0.70		0.71	0.76
11. Easy breathing, respiratory work is not increased	3.21	0.98	4	− 0.05	− 1.04		0.77	0.77
**Capacity to administer oxygen reserves**						0.55		
8. Presence of apnoea or bradycardia with spontaneous recovery, without the carer's intervention	3.86	0.50	4	16.75	− 4.05		0.38	0.77
9. Desaturations	3.82	0.52	4	12.00	− 3.37		0.35	0.77
12. Presence of apnoea or bradycardia with spontaneous recovery, without the carer's intervention	3.87	0.47	4	19.12	− 4.22		0.55	0.78
13. Stable oxygen saturation	3.60	0.86	4	3.72	− 2.22		0.70	0.79
Total						0.78		

### Reliability

The Cronbach's alpha coefficient for the total scale was 0.78 (Table 1). To assess the inter-rater agreement, 36 evaluations were gathered from two independent observers. The overall ICC was 0.94.

### Construct validity

#### Exploratory factor analysis

282 feedings were included. The adequacy of the data was verified using KMO = 0.76 and Bartlett's sphericity test [p < 0.001]. Three factors were identified and explained a total variance of 58.0% (Table 2).

**Table 2:** Exploratory factor analysis of the OFEATINg scale with promax rotation structure matrix.

Items		Communality	F1	F2	F3
Item 1	At the start of feeding, when the teat or nipple rubs the baby's lips, they open their mouth	0.80			0.89
Item 2	When approaching the teat/nipple, the tongue lowers to take it in	0.79			0.88
Item 3	Calm swallowing	0.63	0.79		
Item 4	The newborn loses milk while feeding	0.38	0.60		
Item 5	The newborn stops sucking by themselves to breathe. It is not necessary for the person feeding them to do so	0.66	0.81		
Item 6	Rhythmic sequence and suction fluid	0.59	0.70		
Item 7	Easy breathing, respiratory work is not increased	0.63	0.78		
Item 8	Presence of apnoea or bradycardia with spontaneous recovery, without the carer's intervention	0.75		0.86	
Item 9	Desaturations	0.81		0.90	
Item 10	Is the newborn able to latch on to the teat/nipple?	0.47			0.69
Item 11	Easy breathing, respiratory work is not increased	0.33	0.51		
Item 12	Presence of apnoea or bradycardia with spontaneous recovery, without the carer's intervention	0.31		0.52	
Item 13	Stable oxygen saturation	0.32		0.55	
Percentage of explained variance			29.6	15.7	12.5
Total explained variance		58.0			

F1: factor 1 capacity to coordinate sucking-swallowing-breathing; F2: factor 2 capacity to administer oxygen reserve; F3: factor 3 capacity to take the teat or nipple.

#### Confirmatory factor analysis

**Figure 2:**
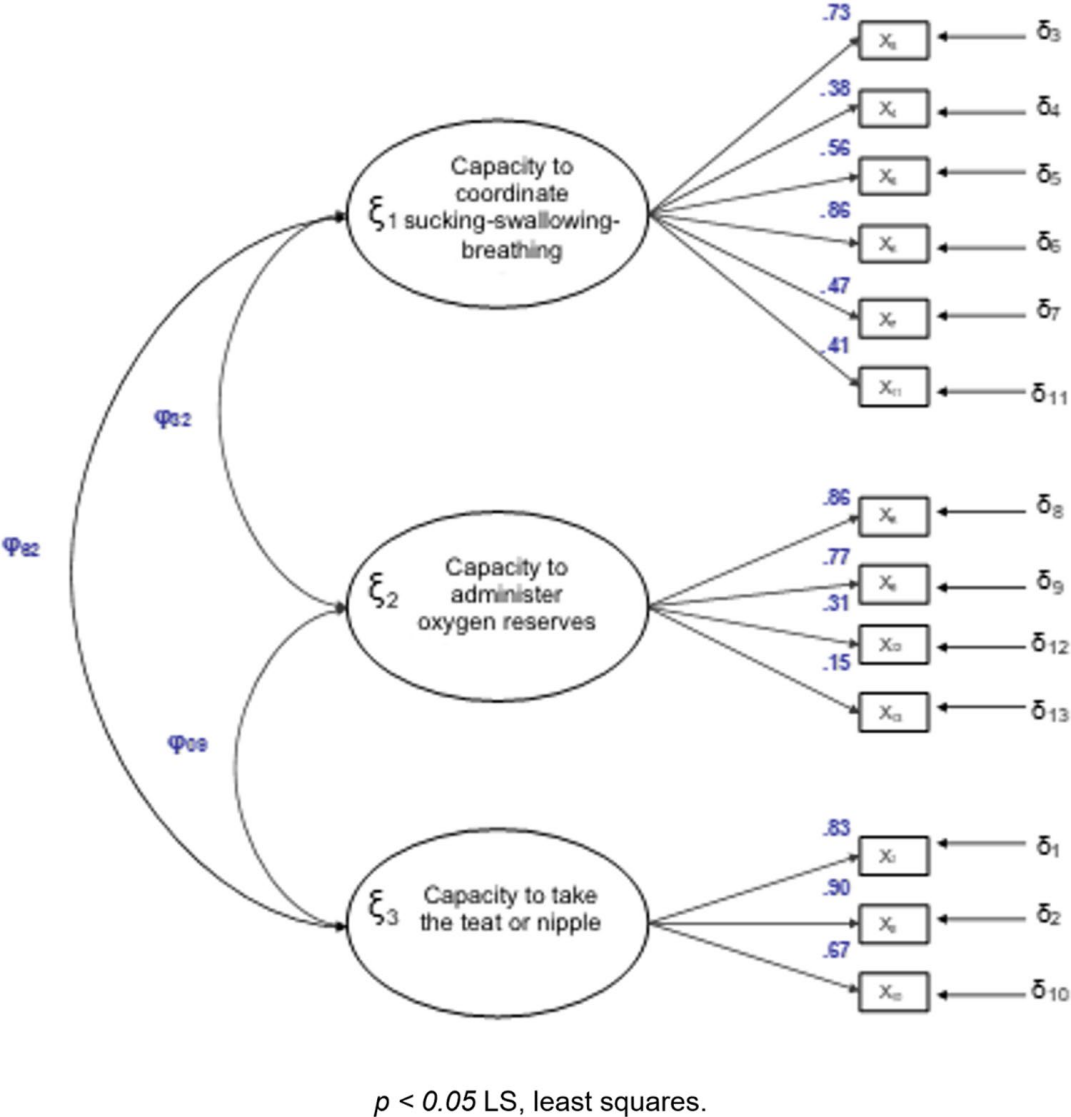
Model specification with 3 factors and 13 indicators.

The model identified in the exploratory factor analysis was used as a base. The indicators of the three factors gave adequate factor loadings. All the saturations were statistically significant. The Chi-squared test gave a p value that indicates statistical significance (χ^2^ = 165.363; gl = 62; *p *= < 0.001, fit χ^2^/gl = 2.66 between 2 and 6). The absolute, incremental and parsimonious fit indices indicated that the model has an adequate fit (Table 3 and Fig. 2).

**Table 3:** Goodness of fit indices of model confirmatory.

Index	Value
BBNFI	0.91
BBNNFI	0.94
CFI	0.95
GFI	0.96
AGFI	0.95
RMSE	0.06
Cronbach’s α	0.78
Goodness of fit test	χ^2^ = 165.363; gl = 62; *p* < .001
Fit reason	χ^2^/gl = 2.66 between 2 and 6

*BBNFI* Bentler–Bonett normed fit index, *BBNNFI* Bentler–Bonett non normed fit index, *CFI* comparative fit index, *GFI* goodness of fit index, *AGFI* adjusted goodness of fit index, *RMSE* root mean standard error, *Df* degrees of freedom.

### Convergent validity

The strongest correlations were observed between the subscales and the total scale. The factor “Capacity to coordinate sucking–swallowing–breathing” shows the strongest correlation with the total scale (r = 0.89), while the weakest correlation is shown by the factor “Capacity to administer oxygen reserves” (r = 0.52) (Table 4).

**Table 4:** Correlations OFEATINg: subscales and total scale.

	Factor 1: capacity to coordinate sucking-swallowing-breathing	Factor 2: capacity to administer oxygen reserves	Factor 3: capacity to take the teat or nipple
Factor 1: capacity to coordinate sucking-swallowing-breathing	1		
Factor 2: capacity to administer oxygen reserves	0.315^a^	1	
Factor 3: capacity to take the teat or nipple	0.379^a^	0.149^a^	1
OFEATINg total scale	0.899^a^	0.523^a^	0.642^a^

^a^All correlations are significant. Significance level: p < 0.01.

### Discriminant validity

Feeding provided without difficulty scored highest on the OFEATINg scale (45 ± 4.4 points compared to 41 ± 4.7 points) with a statistically significant difference of 4.4 points (IC 95%, 3.5–5.3), t(95) = 9.732, *p *< 0.001 (Table 5).

**Table 5:** Evaluation of discriminant validity by comparing means of the OFEATINg scale between the groups that did not have any difficulties during feeding and those that were suspended feeding.

	Mean	SD	Median	Minimum	Maximum	n	p
No difficulties during feeding	45.51	4.49	46	31	52	416	0.0001
Suspended feeding	41.10	4.77	41	29	51	134	
Total feedings	44.44	4.93	45	29	52	550	

### Sensitivity and specificity

**Figure 3:**
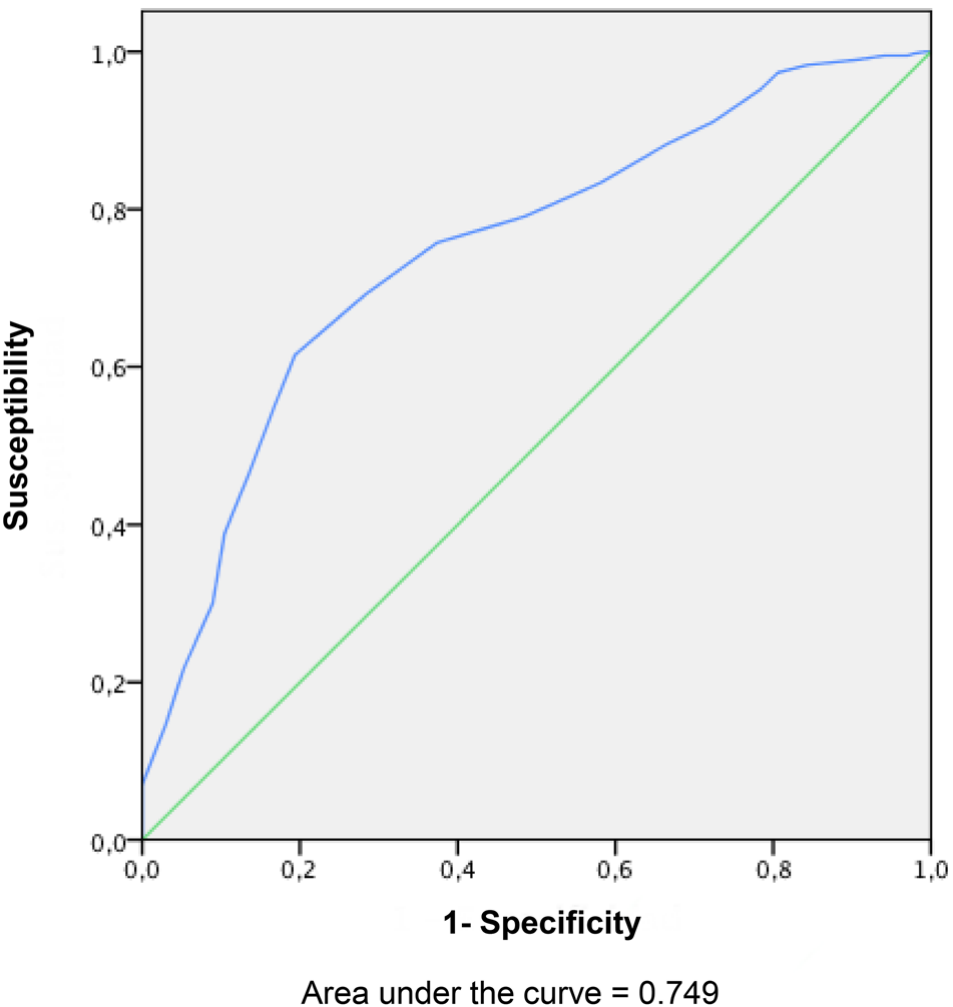
ROC curve obtained from the sum of the instrument scores at each feeding versus the feeding tolerance.

The cut-off points for values above 60 for sensitivity and specificity are 42.5, 43.5 and 44.5. The area under the ROC curve obtained from the sum of the instrument scores for each sample versus the tolerance of the sample was 0.74 (Fig. 3).

## Discussion

The study has allowed us to analyze the measurement properties of the OFEATINg scale. It reports a good internal consistency and three factors that reflect different aspects involved in the oral feeding process.

When comparing the internal consistency of the OFEATINg scale to other instruments, the NOMAS had greater internal consistency, with maximum Cronbach's alpha values of 0.83 in a population of 147 premature infants who were bottle fed^34^. The greater internal consistency of the NOMAS scale may be due to the fact that it assess biomechanical components for successful feeding, like mouth and jaw movements. The reliability of the OFEATINg scale was evaluated on breast and bottle feeds, and its items identified different aspects of oral feeding than the NOMAS scale.

The OFEATINg scale's inter-rater reliability was determined based on feeding observations by healthcare professionals, both in the case of breastfeeding and bottle feeding. The overall Intraclass Correlation Coefficient of the OFEATINg scale was 0.94, ranging between 0.86 and 0.96 in the different factors, which indicates that the instrument has a very good degree of inter-rater agreement. These values are similar to those obtained with the NOMAS scale (0.93- 0.97)^35^.

It is possible to compare the ICC values with the kappa indices obtained in other studies^36^. Therefore, the NOMAS scale obtained kappa indices of 0.40–0.65 and 0.4–0.62^14^, the Preterm Oral Feeding Readiness Assessment Scale (POFRAS)^11^ obtained kappa values of 0.48. Other instruments obtained slightly lower concordance values, such as the NOMAS scale (80.0%)^37^. The OFEATINg scale has a similar level of inter-rater reliability to instruments that only evaluated breastfeeding.

The confirmatory factor analysis enabled us to verify that item 13 had a low factor load (stable oxygen saturation), which suggested that it should be revised in a subsequent version of the scale.

The OFEATINg scale identified three cut off points with sensitivity and specificity above 60% and an area under the ROC curve of 0.74. The only other instrument with this analysis previously was the POFRAS, designed only to evaluate breastfeeding, with slightly lower scores^11,33^.

### Limitations

The inter-rater reliability between professionals and parents is unknown. It has not been possible to evaluate the instrument based on a scale or test that acts as a gold standard.

## Conclusion

Preliminary testing of the OFEATINg scale has shown that is a valid and reliable instrument for evaluating the readiness for oral feeding of preterm infants who are breastfed or bottle fed. In clinical practice, it has the potential to help professionals make decisions involved in the transition of premature infants to full oral feeding. It is easy to learn and administer for nurses working in the NICU. Future lines of research may include clinical validation of the instrument, as well as research comparing strengths and weaknesses of the different instruments and which instruments are preferred by clinicians.

## Data Availability

The data that support the findings of this study are available from Rovira i Virgili University but restrictions apply to the availability of these data, which were used under license for the current study, and so are not publicly available. Data are however available from the authors upon reasonable request and with permission of Rovira i Virgili University.

## Abbreviations

AGFI: Adjusted goodness of fit index
BBNFI: Bentler bonnet normed fit index
BBNNFI: Bentler bonnet non-normed fit index
CFA: Confirmatory factor analysis
EFA: Exploratory factor analysis
EFS: Early feeding skills assessment
GFI: Goodness of fit index
GLS: Generalized least squares
ICC: Intra-class correlation coefficient
KMO: Kaiser–Meyer–Olkin test
NICU: Neonatal intensive care unit
NOMAS: Neonatal oral-motor assessment scale
OFEATINg: Oral feeding assessment in premature infants
PI: Premature infant
RMSE: Root mean standard error
ROC: Receiver operating characteristic
VALOPREM: Valoración de ALimentación Oral en PREMaturos
